# Molecular signature of Epstein Barr virus-positive Burkitt lymphoma and post-transplant lymphoproliferative disorder suggest different roles for Epstein Barr virus

**DOI:** 10.3389/fmicb.2014.00728

**Published:** 2014-12-23

**Authors:** Mohsen Navari, Fabio Fuligni, Maria A. Laginestra, Maryam Etebari, Maria R. Ambrosio, Maria R. Sapienza, Maura Rossi, Giulia De Falco, Davide Gibellini, Claudio Tripodo, Stefano A. Pileri, Lorenzo Leoncini, Pier P. Piccaluga

**Affiliations:** ^1^Hematopathology Section, Department of Experimental, Diagnostic, and Experimental Medicine, S. Orsola-Malpighi Hospital, Bologna University School of MedicineBologna, Italy; ^2^Department of Medical Biotechnology, University of SienaSiena, Italy; ^3^Department of Basic sciences, Torbat Heydariyeh University of Medical SciencesTorbat Heydariyeh, Iran; ^4^Nanchang Joint Programme in Biomedical Sciences, School of Biological and Chemical Sciences, Queen Mary University of LondonLondon, UK; ^5^Microbiology and Virology Unit, Department of Pathology and Diagnostic, University of VeronaVerona, Italy; ^6^Tumour Immunology Unit, Human Pathology Section, Department of Health Science, Palermo University School of MedicinePalermo, Italy

**Keywords:** Epstein Barr Virus, latency, Burkitt lymphoma, post-transplant lymphoproliferative disorder, MicroRNA, gene expression profiling

## Abstract

Epstein Barr virus (EBV) infection is commonly associated with human cancer and, in particular, with lymphoid malignancies. Although the precise role of the virus in the pathogenesis of different lymphomas is largely unknown, it is well recognized that the expression of viral latent proteins and miRNA can contribute to its pathogenetic role. In this study, we compared the gene and miRNA expression profile of two EBV-associated aggressive B non-Hodgkin lymphomas known to be characterized by differential expression of the viral latent proteins aiming to dissect the possible different contribution of such proteins and EBV-encoded miRNAs. By applying extensive bioinformatic inferring and an experimental model, we found that EBV+ Burkitt lymphoma presented with significant over-expression of EBV-encoded miRNAs that were likely to contribute to its global molecular profile. On the other hand, EBV+ post-transplant diffuse large B-cell lymphomas presented a significant enrichment in genes regulated by the viral latent proteins. Based on these different viral and cellular gene expression patterns, a clear distinction between EBV+ Burkitt lymphoma and post-transplant diffuse large B-cell lymphomas was made. In this regard, the different viral and cellular expression patterns seemed to depend on each other, at least partially, and the latency type most probably played a significant role in their regulation. In conclusion, our data indicate that EBV influence over B-cell malignant clones may act through different mechanisms of transcriptional regulation and suggest that potentially different pathogenetic mechanisms may depend upon the conditions of the interaction between EBV and the host that finally determine the latency pattern.

## Introduction

Epstein Barr Virus (EBV) is a human gamma-herpesvirus that normally infects 90–95% of the human normal population, and persists for life of its host. This virus was first discovered in endemic Burkitt Lymphoma (eBL), and was nominated as the first human tumor virus. Today, a couple of human malignancies, other than eBL, are known to harbor the virus, including Post-Transplant Lymphoproliferative Disorder (PTLD) (Leoncini et al., 1993; Bellan et al., 2003; Ghigna et al., 2013).

The manifestation of EBV infection is not the same in all EBV-related tumors, since the virus can adopt different gene expression programs for persistence, called latency types. There are three major types of latencies in EBV-related tumors, which are defined on the basis of the number of EBV latent proteins expressed, with latency type III having the most extensive, where 5 EBV EBNAs (EBV-Nuclear Antigen −1, −2, −3A, −3B, −3C, and -LP) and 3 LMPs (Latent Membrane Proteins −1, −2A, and −2B) are expressed, followed by the less extensive form, i.e., latency type II, where EBNA-1, LMP-1 and LMP-2s are expressed. In latency type I, which is the most limited expression pattern, EBNA-1 molecule is the only latent protein expressed (Gross, 2001; Piccaluga et al., 2011; Onnis et al., 2012).

Furthermore, EBV encodes several non-coding RNA molecules, which include Epstein-Barr virus (EBV)-encoded small RNAs (EBERs) and two different families of microRNAs (miRNAs), non-coding small RNAs with post-transcriptional gene expression regulation ability: BART (BamHI A rightward transcript) and BHRF1 (Bam HI fragment H rightward open reading frame 1). EBERs, similarly to EBNA-1, are expressed in all EBV latency types; the expression of BHRF-1 miRNAs seems to be specific of latency type III while the expression of BART miRNAs is common to all the latency types examined (Pratt et al., 2009; Qiu et al., 2011; Yang et al., 2013; Vereide et al., 2014).

Indeed, the different latency patterns regulate different transcriptional programs as recently shown by Kelly et al. (2013).

Infection of human B cells by EBV *in vitro* results in their constitutive proliferation and immortalization. The resulting cells, called lymphoblastoid cell lines (LCLs), display type III of EBV latency. Although the process of immortalization is not well understood, recent studies focused on the epigenome of different types of human B cells have indicated fundamental differences between LCLs and other type of B-cell, e.g., activated B-cells and BL-cells. (Kreck et al., 2013; Hansen et al., 2014; Hernando et al., 2014) B-cell transformation associated with type III of EBV latency is unlikely to occur in healthy individuals *in vivo* owing to the immunogenic properties of EBNA-2 and -3 proteins which would prompt EBV-infected cell elimination. However, in severely immunocompromised individuals, e.g., organ transplantation, malignant cells similar to LCLs are observed. These malignancies, classified as PTLDs, most commonly are of diffuse large B-cell lymphoma (DLBCL) histotype (Xia et al., 2008; Morscio et al., 2013a,b). A high percentage (70–80%) of PTLDs show positivity for EBV, and the role of the virus seems to be substantial since suppression of the immune system, especially of the cytotoxic T cell branch, leads to higher titers of the virus in the blood, and restoration of the immune response helps the patients to eliminate the tumor (Savoldo et al., 2006). By means of the set of its latent proteins, which are known to be able to deregulate an extensive array of human genes, EBV can contribute to the development of PTLD clones by interfering with different physiologic processes, like proliferation, apoptosis and immune surveillance (reviewed in Morscio et al., 2013b).

Burkitt Lymphoma is subdivided into three subtypes, which consist of the endemic (eBL), sporadic (sBL), and immunodeficiency-related (sometimes called HIV-BL) form. While the majority (90%) of eBLs shows positivity for EBV, the rate of EBV infection in the other two subtypes is infrequent. Of note, an exact role for EBV in eBL lymphomagenesis has not been established yet. However, it has been proposed that there might be a substantial difference in the pathogenetic pathways exerted by EBV in BL-like and LCL-like B cell lymphomas: while the former might primarily depend upon the anti-apoptotic effects of EBV, the driving force for the latter could be the main transforming latent protein of EBV i.e., EBNA-2 (Niller et al., 2004). Furthermore, molecular differences between EBV+ and EBV-negative (EBV−) BL have been reported (Giulino-Roth et al., 2012; Onnis et al., 2012; Shi et al., 2013). Since only one EBV latent protein (EBNA-1) is expressed in BL, a substantial role for EBV-encoded miRNAs in BL has been hypothesized. The expression of EBV miRNAs differs between malignant BL primary tumors and normal B cells from the same patients, being some BART miRNAs absent in non-clonal B cells (Qiu et al., 2011). Furthermore, BART miRNAs have been demonstrated to play a very important role in maintaining the malignant phenotype of BL (Vereide et al., 2014).

Of note, a recent study has addressed the dependency of the malignant phenotype of PTLD and BL cell lines on EBV. Interestingly, a higher level of dependency upon EBV was demonstrated for PTLD cell lines, when compared to BL cell lines (Vereide and Sugden, 2011).

In this work, we aimed at investigating the participation of different latency programs to EBV-related lymphomas at the molecular level. In this regard, we chose to look at EBV-positive (EBV+) BL and EBV+ PTLD_DLBCL as two models representing latency types I and II/III, respectively (i.e., conditions in which EBV oncoproteins are mainly involved or not), and addressed the different contribution of EBV to the transcriptional landscape of these lymphomas by comparing their gene and miRNA expression signatures.

## Experimental procedures

### Ethics statement

The study was conduced in Italy according to the principles of the Helsinki declaration after approval of the Internal Review Board. Written informed consent was obtained from all patients for tissue/cells analysis.

### Study design

We studied a total of 29 EBV+ BL cases, and 28 EBV-positive Post-Transplant Lymphoproliferative Disease (PTLD) morphologically defined as diffuse large B-cell lymphomas (DLBCL). Briefly, DLBCL cases presented with either early (*N* = 10) or late (*N* = 13) onset, and EBV latency type II (*N* = 8) or III (*N* = 13) or not defined (*N* = 2). Details on such cases are reported in Supplementary Data Sheet 1.

**Figure 1:**
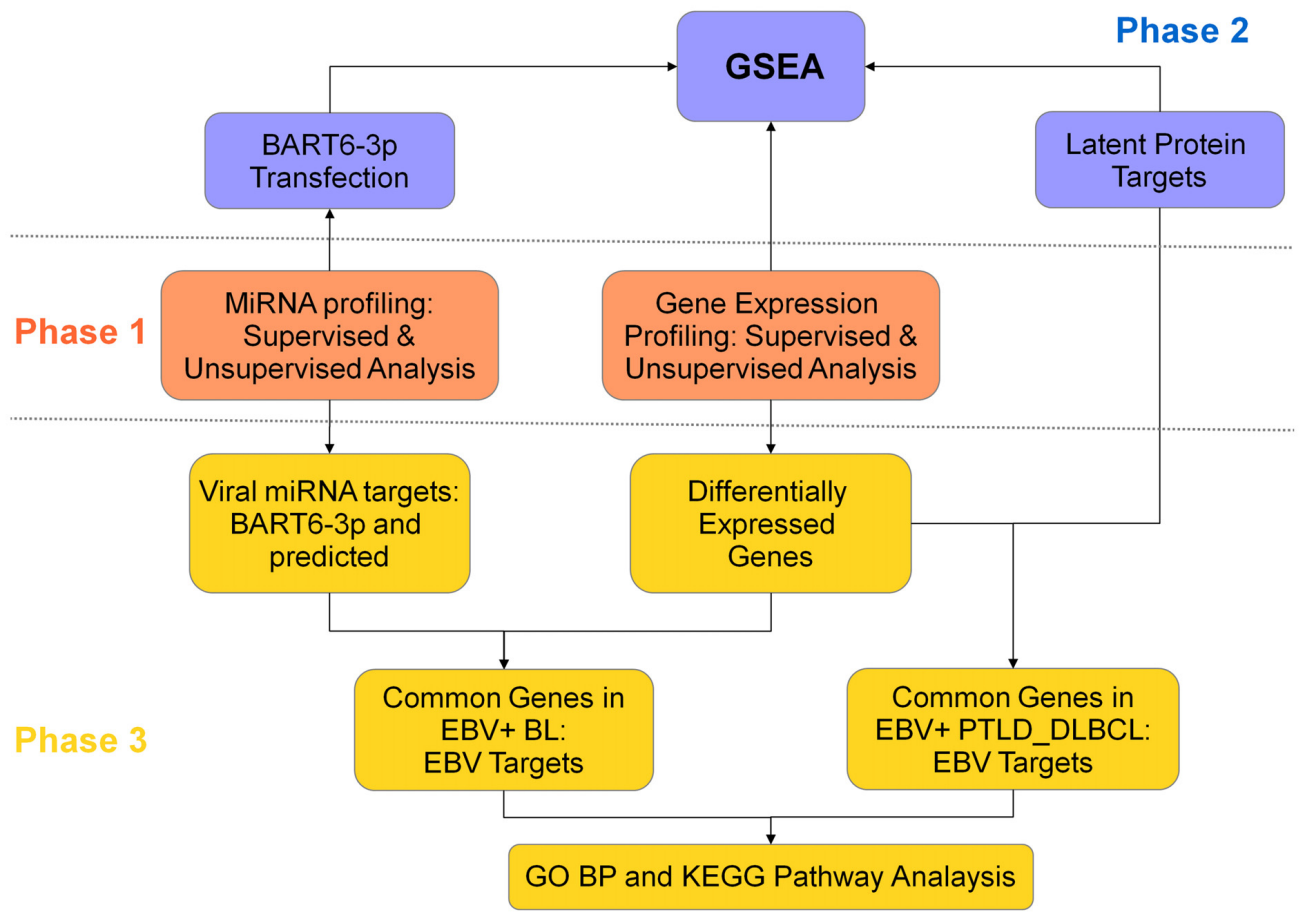
**Study design**. This project, with goal of comparing the role of EBV in EBV+ BL and EBV+ PTLD_DLBCL, was designed in three phases. In the first phase, the molecular signatures of the two tumors were compared for both gene expression and miRNA profiling (middle panel, orange). In the second phase, the role of viral latent proteins and EBV-encoded miRNA ebv-miR-BART6-3p was evaluated, by usage of Gene Set Enrichment Analysis (top panel, violet). In the third phase, the differentially expressed genes in the two tumors were coupled to the corresponding miRNA, in EBV+ BL, or to latent proteins, in EBV+ PTLD_DLBCL. The extracted genes were further analyzed in the terms of biological processes and pathways (bottom panel, yellow).

This study was designed in three phases (Figure 1). In the first phase, we compared the miRNA and gene expression profile (GEP) of EBV+ BL and EBV+ PTLD_DLBCL, in order to evaluate their similarities/differences. Second phase was aimed to explore the effect of EBV on the global gene expression of the two tumors. For this purpose, by means of Gene Set Enrichment Analysis (GSEA), the expression of the genes deregulated by EBV latent proteins (extracted from the literature), or the genes deregulated by one of EBV-encoded miRNAs, i.e., ebv-miR-BART6-3p (which were determined experimentally) was evaluated in EBV+ BL and EBV+ PTLD_DLBCL. In the third phase, we looked for the genes targeted by EBV latent proteins and miRNAs among genes differentially regulated in the considered tumor categories and looked for their enrichment in biological processes and pathways using Molecular Signature DataBase (MsigDB) web-based tool, as describer later.

An analog approach, except for the third phase, was used to investigate possible differences between EBV+ PTLD_DLBCLs characterized by latency types II or III.

### Cell transfection and microarray hybridization

In order to determine the ebv-miR-BART6-3p targets, EBV-negative Akata 2A8 or EBV+ Akata cells were transiently transfected with synthetic ebv-miR-BART6-3p mimic or inhibitor (100 nM; Custom synthesized by Dharmacon- Thermo Scientific, Germany), respectively. For each treatment, a further transfection with corresponding negative control was performed as well (10 nM; I-300145-01, Dharmacon- Thermo Scientific, Germany). The transfection of 5 × 10^6^ cells per treatment was performed by Amaxa nucleofector apparatus (Amaxa, Cologne-Germany), using the program G23 and the transfection solution V according to the manufacturer's instructions. Transfection efficiency was assessed by means of a further treatment (2 μg of pmaxGFP) and detection of both fluorescence and cell viability by flow cytometry. Twenty four hours post-transfection, cells were harvested and RNA was extracted using Trizol, and transfection efficiency was further confirmed by evaluating the expression level of ebv-miR-BART6-3p using q-PCR by means of Taqman probes, employing RNU43 as housekeeping miRNA (Applied Biosystems, Cologne, Germany), as described (Onnis et al., 2012). RNA was further hybridized to HuGene-2.0-st array (Affymetrix, Santa Clara, CA) according to the manufacturer's instructions. The resulting data are available online at the Gene Expression Omnibus (www.ncbi.nlm.nih.gov/projects/geo/), accession number GSE63665.

### Viral miRNA target prediction

The prediction of viral miRNAs targets was accomplished using Viral microRNA Host Target Database (vHoT DB), which collects the target predictions from 5 publicly available databases, including TargetScan, miRanda, RNAhybrid, DIANA microT, and PITA (Kim et al., 2012). The variables were set as follows: Minimum Free Energy (MFE) threshold values for miRanda and RNAhybrid were set to −30 and −40 kcal/mol, respectively; Total Context Score threshold value for Targetscan was set to −1, and the Score threshold value for DIANA microT and ddG threshold value for PITA were set to the default. For each miRNA, the targets that were found in at least 3 out of 5 databases were chosen.

### MicroRNA expression profiling analysis

MicroRNA expression profiling was performed on 17 cases of EBV+ BL and 5 cases of EBV+ PTLD_DLBCL, for which Formalin Fixed Paraffin Embedded (FFPE) tissues were available in the archives of the Hematopathology Unit in Bologna and the Pathology Unit in Siena (Supplementary Data Sheet 2), using the nCounter® miRNA Expression Assay Kits (NanoString Technologies, Seattle, WA, USA). Raw data coming from nCounter® miRNA Expression Assay were normalized using NanoStringNorm package developed in R 2.15 version. Briefly, probe levels quantified by microarrays were adjusted for miRNAs with specific background correction factor. Technical normalization was performed using geometric mean of positive controls and mean of negative controls for background subtraction. Lastly, the dataset was normalized such that the mean of each gene is zero. The data were further analyzed in the terms of Unsupervised Hierarchical Clustering Analysis (HCA) and Principal Component Analysis (PCA) using GeneSpring version (Agilent Technologies), as described (Piccaluga et al., 2007, 2008, 2011). The miRNAs differentially expressed among the two categories were selected on the basis of the following criteria: fold change ≥2, corrected *p*-value (Benjamini-Hockeberg FDR) ≤0.05.

### Gene expression profiling analysis

For gene expression profiling, the CEL files of 23 cases of EBV+ PTLD_DLBCL (Supplementary Data Sheet 1) and 12 cases of EBV+ BL were extracted from Gene Omnibus Database (GSE38885, GSE26673; Piccaluga et al., 2011; Morscio et al., 2013a). The CEL files were quantile normalized and log2 transformed using rma method implemented by means of affy Bioconductor R package. When comparing EBV+ PTLD_DLBCL cases with EBV+ BLs, the resulting matrix was further normalized for batch effect by means of ComBat package in R. Furthermore, in all cases a further step of mean-variance normalization was achieved using geWorkbench 2.4.1. Unsupervised HCA was performed using MeV 4.9 software. Because of memory constraints of this software, the top 7 × 10^3^ probes in the terms of highest standard deviation were chosen. Unsupervised PCA was done by means of R software. Supervised analysis (two-tails student *T*-test) was performed to obtain molecular signatures of different tumors using geWorkbench 2.4.1 software and filtered using the following criteria: *p*-value ≤0.05, fold change ≥2. When comparing EBV+ BL vs. EBV+ PTLD_DLBCL, the analysis was also performed by filtering the resulting probes against those characterizing the molecular signature generically discriminating BL and DLBCL described by Hummel et al. (2006). The differentially expressed probes were further used to perform supervised HCA using MeV 4.9 software. During all the analysis steps, except for when comparing EBV+ BL against the whole set of EBV+ PTLD_DLBCLs, the two EBV+ PTLD_DLBCL cases for which the latency type was not available were omitted.

The CEL files resulting from ebv-miR-BART6-3p transfection experiments were normalized and analyzed as described above, except for ComBat normalization, which was omitted in this case.

### Identification of EBV target genes

To identify the possible targets of EBV, we looked for the predicted and experimentally established targets of EBV-encoded products in the genes differentially expressed in the two tumor categories.

For the potential target genes of latent proteins, we used the previously published data, which addressed the effect of EBV latent proteins on the molecular profile of the cells, as a source of target genes. These studied latent proteins included EBNA-1 (Dresang et al., 2009), EBNA-2 (Maier et al., 2006), EBNA-3 proteins (EBNA-3s, White et al., 2010) and LMP-1 (Vockerodt et al., 2008). In the case of EBNA-3s, we decided to consider the effect of their simultaneous expression, since a possible collaboration among them has been proposed (White et al., 2010). Furthermore, we excluded EBNA-LP, since the only study that has addressed its effect on the—global gene signature of human cells, gave result to a too small number of genes to be used for GSEA (Kanamori et al., 2004). Whenever possible, the genes with the following criteria were chosen: *p*-value ≤0.05, fold change ≥2. We then looked for these target genes among the genes differentially expressed in EBV+ PTLD_DLBCL vs. EBV+ BL, taking a positive correlation approach, i.e., the genes that were up-regulated by latent proteins were searched for in those up-regulated in EBV+ PTLD_DLBCL and vice versa.

In the case of EBV-encoded miRNAs, we took into account both experimentally found targets (i.e., those resulting from the ebv-miR-BART6-3p transfection experiment described in this paper) and those predicted by at least 3 prediction algorithms (described above). We then searched for these targets in the genes differentially expressed in the considered tumor categories, keeping a negative correlation manner, i.e., targets of up-regulated miRNAs in a tumor were looked for in the genes down-regulated in that tumor and vice versa.

Furthermore, the common potential target genes of either miRNAs or viral latent proteins down-regulated in a tumor entity and up-regulated in the other were identified.

### Gene set enrichment analysis (GSEA)

Enrichment in expression of EBV target genes was evaluated using Gene set enrichment analysis (GSEA) software on the set samples described above (EBV+ BL vs. EBV+ PTLD_DLBCL or EBV+ PTLD_DLBCL of latency type II vs. EBV+ PTLD_DLBCL of latency type III).

GSEA of the genes potentially deregulated by EBV was performed in the terms of Gene Ontology Biological Process and Kyoto Encyclopedia of Genes and Genomes (KEGG) pathways by means of GSEA MsigDB (www.broadinstitute.org/gsea/msigdb) web-based analysis tool, using the default options (displaying top 10 gene sets with FDR *q*-value below 0.05).

## Results

### EBV+ BL and PTLD_DLBCL have different molecular profiles

To unveil the possible molecular differences/similarities between EBV+ BL and EBV+ PTLD_DLBCL, we performed a comparison of the expression profile of both genes and miRNAs between the two entities. In particular, for GEP we used 12 cases of EBV+ BL and 23 cases of EBV+ PTLD_DLBCL which were previously generated and available at GEO database. For miRNA profiling, including both viral and cellular miRNAs, we used 17 EBV+ BL and 5 EBV+ PTLD_DLBCL cases that were profiled by Nanostring technology.

**Figure 2:**
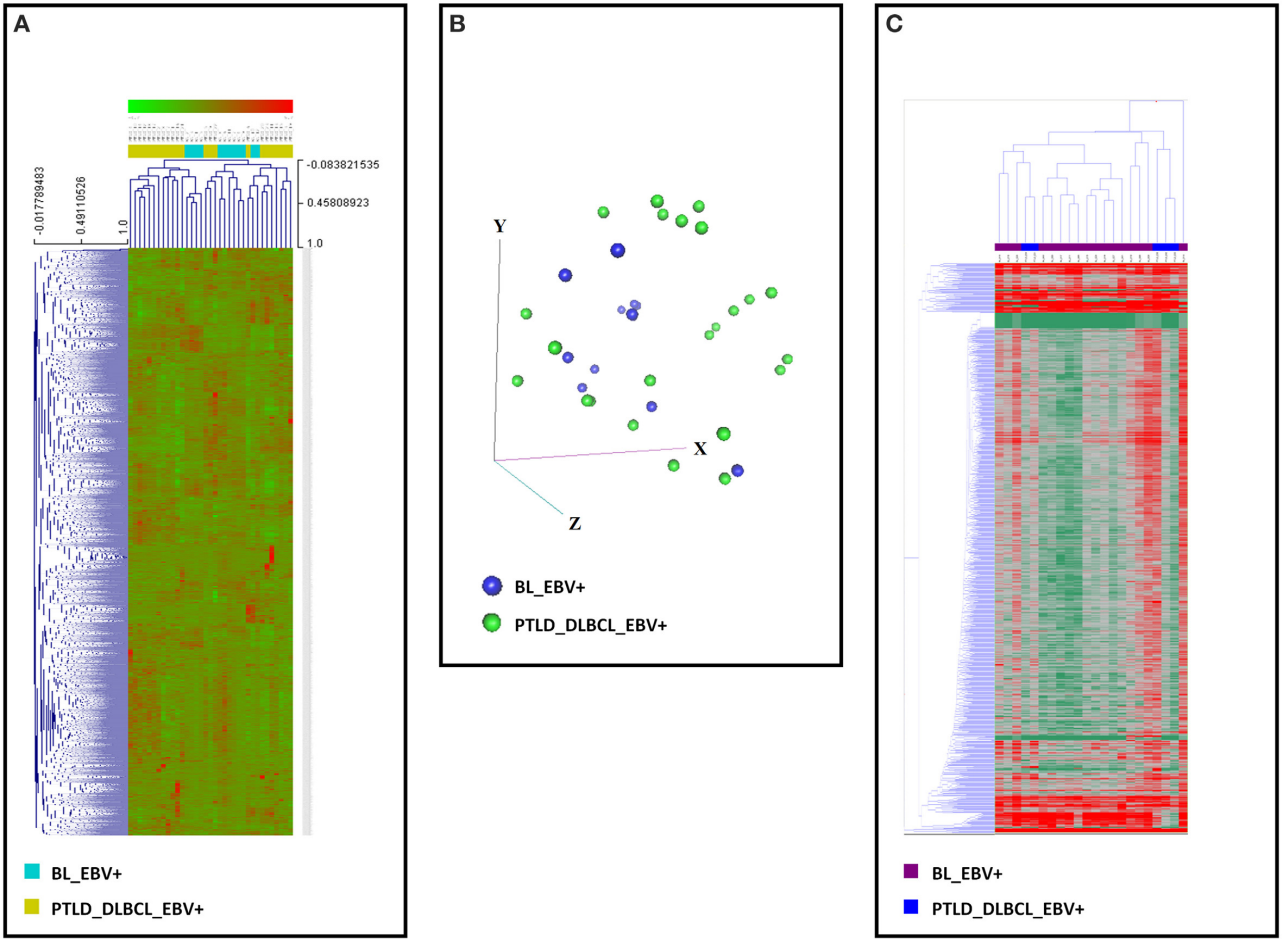
**Gene and miRNA expression analysis of EBV+ BL and EBV+ PTLD_DLBCL**. Unsupervised analyses failed to distinguish the two tumor entities. **(A)** Unsupervised hierarchical clustering based on gene expression is depicted. **(B)** Principal Component Analysis showed a significant overlap of the two tumor entities. The three main components (i.e., groups of genes) are represented in the three axes (x, y, and z). **(C)** Unsupervised hierarchical clustering based on the expression of viral and cellular miRNA was again unable to discriminate the two entities. Together, these data indicate that EBV+ BL and EBV+ PTLD_DLBCL present significant similarities in both their gene and miRNA expression profiles. In the heat-map each row represents a gene **(A)** or miRNA **(C)** and each column represents a sample. The color scale illustrates the relative expression level of a gene across all samples: red represents an expression level above the mean, green represents expression lower than the mean.

The unsupervised analysis, i.e., unsupervised HCA and PCA, did not allow to clearly discriminate between the two categories on both gene (Figures 2A,B) and miRNA profiling (Figure 2C).

In order to identify possible differences between the tumors, a supervised test was then performed. As far as GEP was concerned, we found 780 probes significantly up-regulated in EBV+ BLs, and 1247 probes significantly up-regulated in EBV+ PTLD_DLBCLs, which corresponded to 513 and 801 unique genes, respectively (Supplementary Data Sheet 3).

Regarding the miRNA profiling, on the other hand, we found 36 miRNA differentially expressed: 21 of these miRNAs were over-expressed in EBV+ BLs and most (*n* = 17) consisted of EBV-encoded miRNAs. The over-expressed miRNAs in EBV+ PTLD_DLBCLs (*n* = 14) were almost entirely cellular miRNAs with the exception of one EBV-encoded miRNA, i.e., ebv- miRBHRF-1-2 (Table 1).

**Table 1:** **Differentially expressed miRNAs in EBV+ BL and EBV+ PTLD_DLBCL**.

**MicroRNA**	***P*-value**	**FC (abs)**	**Regulation**	**Accession**
ebv-miR-BART19-3p	5.04E-005	7.3412666	Up-regulated in EBV+ BL	nmiR00733.1
			(Down-regulated in EBV+ PTLD_DLBCL)	
ebv-miR-BART17-3p	5.57E-004	9.606497	Up-regulated in EBV+ BL	nmiR00729.1
			(Down-regulated in EBV+ PTLD_DLBCL)	
hsa-miR-92a	6.75E-004	2.9477286	Up-regulated in EBV+ BL	nmiR00668.1
			(Down-regulated in EBV+ PTLD_DLBCL)	
ebv-miR-BART22	0.00101368	14.518561	Up-regulated in EBV+ BL	nmiR00741.1
			(Down-regulated in EBV+ PTLD_DLBCL)	
ebv-miR-BART8	0.00143009	5.792101	Up-regulated in EBV+ BL	nmiR00748.1
			(Down-regulated in EBV+ PTLD_DLBCL)	
ebv-miR-BART3	0.00156815	8.551074	Up-regulated in EBV+ BL	nmiR00742.1
			(Down-regulated in EBV+ PTLD_DLBCL)	
ebv-miR-BART9	0.001634	8.696155	Up-regulated in EBV+ BL	nmiR00749.1
			(Down-regulated in EBV+ PTLD_DLBCL)	
ebv-miR-BART1-3p	0.00232697	6.0770316	Up-regulated in EBV+ BL	nmiR00719.1
			(Down-regulated in EBV+ PTLD_DLBCL)	
ebv-miR-BART4	0.00274992	4.232079	Up-regulated in EBV+ BL	nmiR00743.1
			(Down-regulated in EBV+ PTLD_DLBCL)	
ebv-miR-BART11-5p	0.00428005	5.031375	Up-regulated in EBV+ BL	nmiR00723.1
			(Down-regulated in EBV+ PTLD_DLBCL)	
ebv-miR-BART19-5p	0.00479706	5.03283	Up-regulated in EBV+ BL	nmiR00734.1
			(Down-regulated in EBV+ PTLD_DLBCL)	
hsa-miR-484	0.00649317	2.6175013	Up-regulated in EBV+ BL	nmiR00385.1
			(Down-regulated in EBV+ PTLD_DLBCL)	
hsa-miR-1274b	0.00677765	2.1696913	Up-regulated in EBV+ BL	nmiR00093.1
			(Down-regulated in EBV+ PTLD_DLBCL)	
kshv-miR-K12-6-5p	0.00678478	2.0836391	Up-regulated in EBV+ BL	nmiR00795.1
			(Down-regulated in EBV+ PTLD_DLBCL)	
ebv-miR-BART21-3p	0.00949766	3.7267542	Up-regulated in EBV+ BL	nmiR00739.1
			(Down-regulated in EBV+ PTLD_DLBCL)	
ebv-miR-BART10	0.01241677	3.8621666	Up-regulated in EBV+ BL	nmiR00721.1
			(Down-regulated in EBV+ PTLD_DLBCL)	
ebv-miR-BART6-3p	0.01335738	3.816174	Up-regulated in EBV+ BL	nmiR00745.1
			(Down-regulated in EBV+ PTLD_DLBCL)	
ebv-miR-BART12	0.01714046	3.8982465	Up-regulated in EBV+ BL	nmiR00724.1
			(Down-regulated in EBV+ PTLD_DLBCL)	
ebv-miR-BART6-5p	0.01769217	3.296042	Up-regulated in EBV+ BL	nmiR00746.1
			(Down-regulated in EBV+ PTLD_DLBCL)	
hsa-miR-20a+ hsa-miR-20b	0.01775939	23.560373	Up-regulated in EBV+ BL	nmiR00236.1
			(Down-regulated in EBV+ PTLD_DLBCL)	
ebv-miR-BART17-5p	0.02827049	2.9462714	Up-regulated in EBV+ BL	nmiR00730.1
			(Down-regulated in EBV+ PTLD_DLBCL)	
ebv-miR-BART7	0.0311731	3.0952694	Up-regulated in EBV+ BL	nmiR00747.1
			(Down-regulated in EBV+ PTLD_DLBCL)	
hsa-miR-155	1.03E-006	9.04388	Up-regulated in EBV+ PTLD_DLBCL)	nmiR00178.1
			(Down-regulated in EBV+ BL)	
hsa-miR-630	3.43E-004	8.03204	Up-regulated in EBV+ PTLD_DLBCL)	nmiR00586.1
			(Down-regulated in EBV+ BL)	
hsa-miR-1978	6.99E-004	3.3640883	Up-regulated in EBV+ PTLD_DLBCL)	nmiR00694.1
			(Down-regulated in EBV+ BL)	
hsa-miR-211	0.00150208	4.2735744	Up-regulated in EBV+ PTLD_DLBCL)	nmiR00240.1
			(Down-regulated in EBV+ BL)	
hsa-miR-663b	0.00248701	3.742663	Up-regulated in EBV+ PTLD_DLBCL)	nmiR00621.1
			(Down-regulated in EBV+ BL)	
hsa-miR-494	0.00359945	149.04005	Up-regulated in EBV+ PTLD_DLBCL)	nmiR00400.1
			(Down-regulated in EBV+ BL)	
hsa-miR-1973	0.00667112	2.7127783	Up-regulated in EBV+ PTLD_DLBCL)	nmiR00689.1
			(Down-regulated in EBV+ BL)	
hsa-miR-21	0.01024776	2.768076	Up-regulated in EBV+ PTLD_DLBCL)	nmiR00238.1
			(Down-regulated in EBV+ BL)	
hsa-miR-24	0.01031194	2.183924	Up-regulated in EBV+ PTLD_DLBCL)	nmiR00261.1
			(Down-regulated in EBV+ BL)	
hsa-miR-1308	0.01368784	3.4283412	Up-regulated in EBV+ PTLD_DLBCL)	nmiR00131.2
			(Down-regulated in EBV+ BL)	
hsa-miR-222	0.01613653	2.0202823	Up-regulated in EBV+ PTLD_DLBCL)	nmiR00256.1
			(Down-regulated in EBV+ BL)	
hsv1-miR-H8	0.03170305	2.972884	Up-regulated in EBV+ PTLD_DLBCL)	nmiR00779.1
			(Down-regulated in EBV+ BL)	
ebv-miR-BHRF1-2	0.03283407	3.6006873	Up-regulated in EBV+ PTLD_DLBCL)	nmiR00751.1
			(Down-regulated in EBV+ BL)	
hsa-miR-330-5p	0.03623376	2.563781	Up-regulated in EBV+ PTLD_DLBCL)	nmiR00307.1
			(Down-regulated in EBV+ BL)	

**Figure 3:**
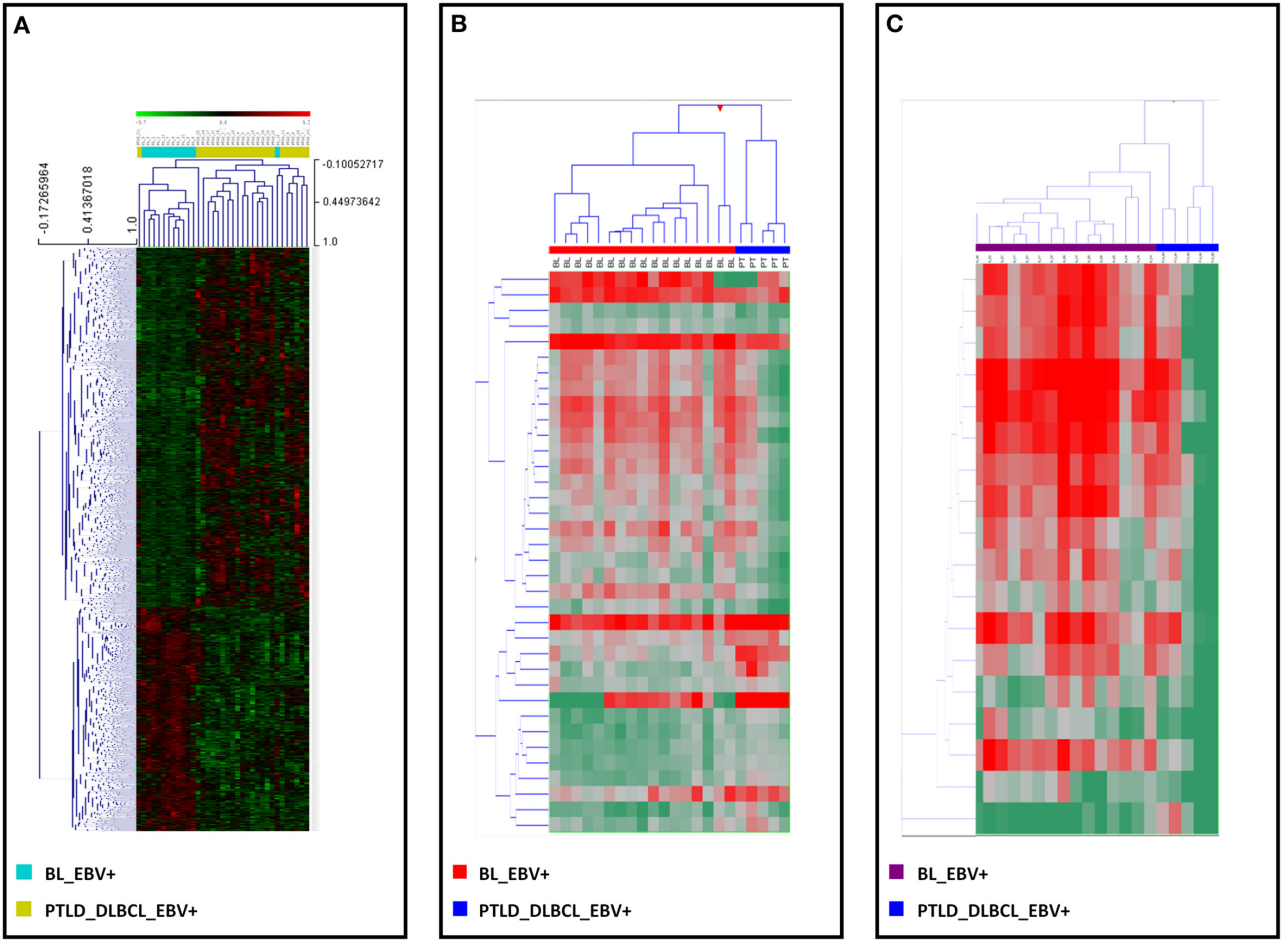
**EBV+ BL and EBV+ PTLD_DLBCL have different molecular profiles**. Supervised analysis identified genes and miRNAs differentially expressed in EBV+ BL vs. EBV+ PTLD_DLBCL. **(A)** Supervised hierarchical clustering based on the differentially expressed genes is shown. **(B)** Supervised hierarchical clustering based on the differentially expressed miRNAs, including both viral and cellular miRNAs is shown. **(C)** Supervised hierarchical clustering based on the differentially expressed viral only miRNAs is presented. In the heat-map each row represents a gene **(A)** or miRNA **(B,C)** and each column represents a sample. The color scale illustrates the relative expression level of a gene across all samples: red represents an expression level above the mean, green represents expression lower than the mean.

Of note, following recognition of the differentially expressed genes and miRNAs, a supervised HCA led to a clear distinction between the two, for both genes (Figure 3A) and miRNAs (Figure 3B). Interestingly, when examining differentially expressed viral miRNAs only (excluding cellular miRNAs), it was still possible to efficiently discriminate the two tumor entities (Figure 3C).

### EBV-encoded latent proteins might significantly affect the molecular signature of EBV+ PTLD_DLBCL

To investigate how the latent proteins encoded by EBV, which are predominantly expressed in EBV+ PTLD_DLBCL rather than in BL, might impact the molecular profile of our entities, we examined the expression of their target genes. In particular, we focused on the genes the expression of which was regulated by EBNA-1, EBNA-2, EBNA-3s and LMP-1, and by means of GSEA, we tested their expression in EBV+ BL vs. EBV+ PTLD_DLBCL.

**Figure 4:**
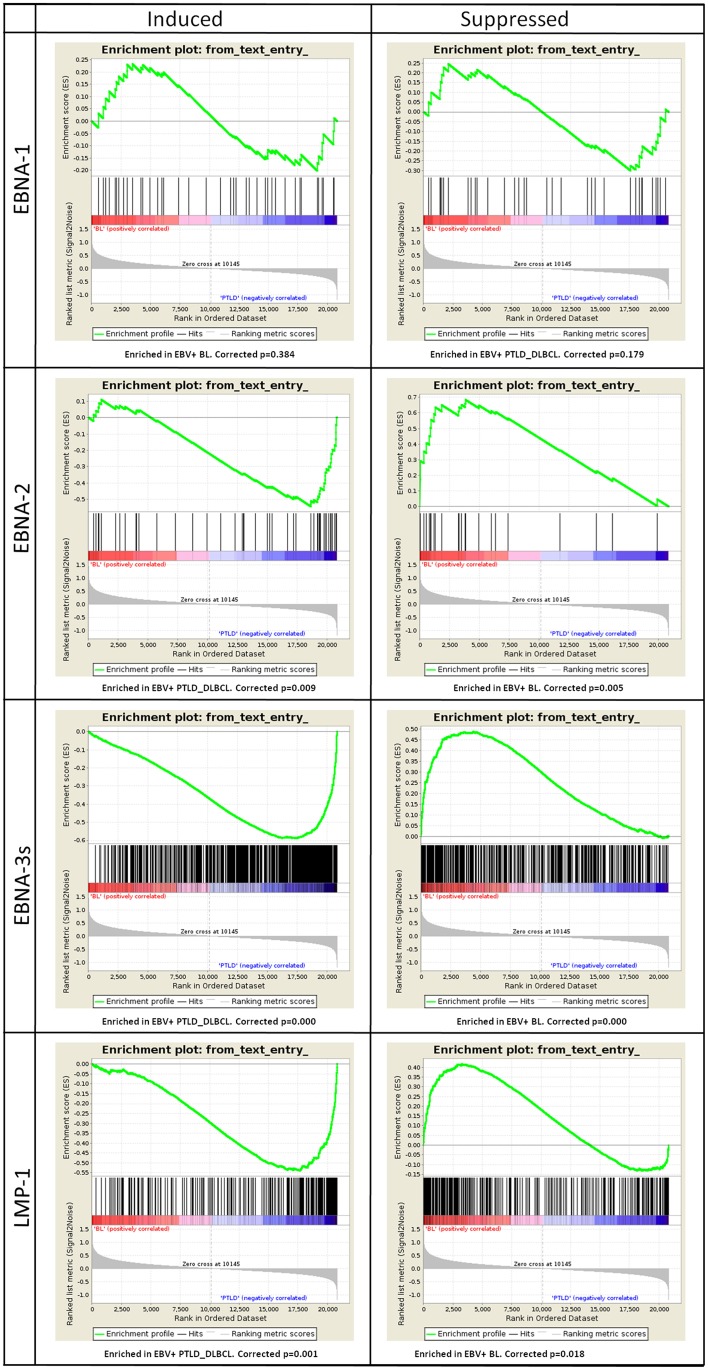
**Gene Set Enrichment Analysis (GSEA) of EBV latent proteins-dependent signatures reveals their effect on the molecular profiles of EBV+ PTLD_DLBCL**. The effect of viral latent proteins expressed across the different latency programs were investigated in EBV+ PTLD_DLBCL and EBV+ BL. GSEA was applied to study the expression of target genes of the latent proteins of EBV in the two tumor types. For each latent protein (EBNA-1, EBNA-2, EBNA-3s, and LMP-1) induced or suppressed targets were analyzed (left and right, respectively). While EBNA-1 targets were not significantly affected, as expected by the latency program, EBNA-2, EBNA-3s, and LMP-1 targets appeared to be significantly enriched in either one group. Particularly, induced targets were enriched in EBV+ PTLD_DLBCL and the suppressed ones were enriched in EBV+ BL.

The genes induced (*n* = 44) or suppressed by EBNA-1 (*n* = 36) resulted in no significant enrichment (corrected *p*-value equal to 0.384 and 0.179 for induced and suppressed genes, respectively). Conversely, the genes induced by EBNA-2 (*n* = 52), EBNA-3s (*n* = 810) and LMP-1 (*n* = 249) all turned out to be significantly enriched in EBV+ PTLD_DLBCL, with high confidence (corrected *p*-value equal to 0.009, 0.000, and 0.0001, correspondingly). Accordingly, the genes whose expression was suppressed by EBNA-2 (*n* = 23), EBNA-3s (*n* = 483) and LMP-1 (*n* = 308) were all clearly enriched in EBV+ BL (corrected *p*-value equal to 0.005, 0.000 and 0.018, respectively) (Figure 4).

Together, these results provide strong evidence that different EBV latent protein expression profiles influence the transcriptional programs of EBV-associated BL- and PTLD_DLBCL-cells. Moreover, they indicate that EBV largely affects PTLD gene expression profile mainly through viral latent protein expression, while little of this viral latent protein activity is exerted in BL.

### ebv-miR-BART6-3p, an EBV-encoded miRNA, can influence the molecular profile of EBV+ BL

**Figure 5:**
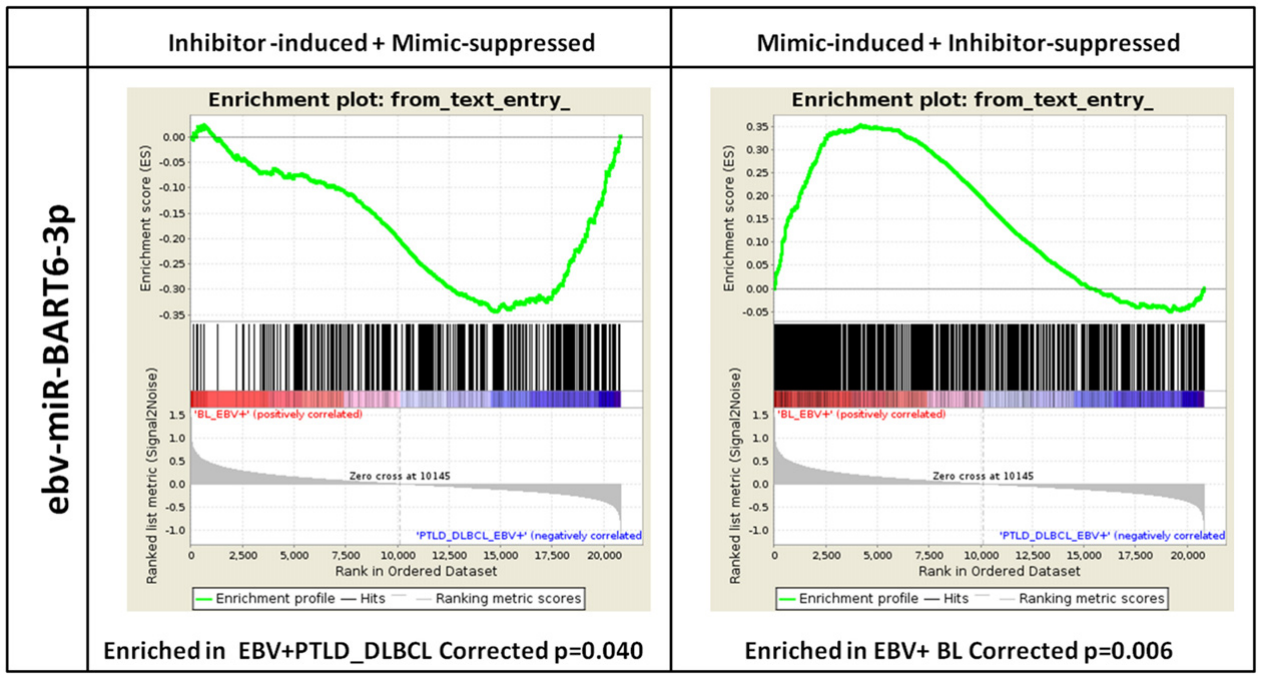
**ebv-miR-BART6-3p expression can affect the molecular profile of EBV+ BL**. Based on the number and biological importance of its predicted target genes, ebv-BART6-3p was chosen as a proof of principle for investigating the potential effect of viral miRNAs in EBV+ BL. Akata 2A8 (EBV−) and Akata (EBV+) cells were transfected with ebv-BART6-3p mimic or inhibitor, respectively, along with the corresponding controls, and gene expression analysis was performed by Affymetrix HuGene 2.0 microarray. Genes up-regulated upon ebv-BART6-3p inhibition and down-regulated upon ebv-BART6-3p induction were significantly enriched in EBV+ PTLD_DLBCL cases (left panel). Conversely, genes down-regulated upon ebv-BART6-3p inhibition and up-regulated upon ebv-BART6-3p induction were significantly enriched in BL (right panel). Both results were then consistent with ebv-BART6-3p expression and active role in BL.

In order to evaluate the impact of the differentially expressed EBV-encoded miRNAs on the global GEP of the tumors, we then selected a model that served as proof of principle. Specifically, we chose ebv-miR-BART6-3p, as it turned out to be among the top 3 viral miRNAs in the terms of the highest number of target genes predicted by conventional bioinformatics tools (Supplementary Data Sheet 4) and its predicted effects could be biologically sound. We then transfected Akata 2A8 (EBV−) and Akata (EBV+) cells by either mimic or inhibitor of ebv-miR-BART6-3p, respectively, and generated gene expression profiles of the treated cells. Ectopic expression of ebv-miR-BART6-3p led to induction of 593 and suppression of 724 probes, while in the case of knocking down this miRNA, we found 861 probes under-expressed and 1074 probes over-expressed (Supplementary Data Sheet 5, 6). Totally, we detected 962 and 746 unique genes to have either positive or negative correlation with ebv-miR-BART6-3p expression, respectively (Supplementary Data Sheet 7). Interestingly, when examined using GSEA, genes with negative correlation with ebv-miR-BART6-3p were significantly enriched in EBV+ PTLD_DLBCLs (*p* = 0.040), while those with a positive correlation were significantly enriched in EBV+ BLs (*p* = 0.006, Figure 5). This result proved that BL and PTLD differentially modulate *in vivo* (i.e., in primary tumors) genes targeted by differentially expressed viral miRNAs (e.g., ebv-miR-BART6-3p) and indicated that miRNA expression is the primary mechanism through which EBV does influence the molecular profile of BL cells.

### EBV-deregulated genes in different EBV latency types might interfere with relevant biological activities

As it appeared that EBV significantly affected, though in different ways, the global GEP of both BL and PTLD-DLBCL, we investigated whether genes likely to be modulated by EBV were involved in specific biological functions, aiming to further appreciate its specific role in the pathobiology of these lymphomas. In particular, we evaluated the genes regulated by EBNA-2, EBNA-3s, LMP-1, the ones predicted to be targets of the EBV-encoded miRNAs as well as those induced and suppressed by ebv-miR-BART6. In addition, we looked for EBV target genes that might be induced in one tumor group and suppressed in the other group by different mechanisms.

Specifically, 174 (21.7%) of the genes up-regulated and 61 (11.8%) of the genes down-regulated in EBV+ PTLD_DLBCL vs. EBV+ BL were found to be potentially induced or suppressed by latent proteins, respectively (Supplementary Figures 1A and 1B, Supplementary Data Sheet 8, 9). On the other hand, 113 (14%) of the genes down-regulated and 21 (4%) of the genes up-regulated in EBV+ BL were found likely to be regulated by differentially expressed BART miRNAs (including ebv-miR-BART6-3p) or ebv-miR-BART6-3p, accordingly (Supplementary Figures 1C,D, Supplementary Data Sheet 10, 11). Furthermore, we identified 38 common target genes that could potentially be repressed by EBV miRNAs in EBV+ BL, or conversely, be induced by latent proteins in EBV+ PTLD_DLBCL (Supplementary Figure 1E, Supplementary Data Sheet 12).

The potential role of such modulated genes was investigated looking at their enrichment in GO biological processes and KEGG pathways. It was found that the genes purportedly up-regulated by EBV latent proteins in EBV+ PTLD_DLBCL were significantly enriched in functions such as apoptosis, immune response and developmental regulation (Supplementary Figure 2A), while the genes down-regulated by latent proteins in this tumor were enriched in processes especially related to RNA metabolism, including transcription, regulation of gene expression, and RNA biosynthesis-related processes (Supplementary Figure 2B). On the other hand, the genes possibly suppressed by EBV-encoded miRNAs in EBV+ BL were significantly enriched in regulation of different biological processes like signal transduction, gene expression and metabolism (Supplementary Figure 2C). Furthermore, possible common target genes of EBV suppressed in EBV+ BL and induced EBV+ PTLD were significantly enriched in regulation of apoptosis, immune system process, humoral immune response, and regulation of different biological processes (signal transduction, gene expression and metabolism), (Supplementary Figure 2D). The ebv-miR-BART6-3p targets induced in EBV+ BL were not meaningfully enriched in any GO processes.

Concerning the enrichment of our gene sets in KEGG pathways, for the potential targets of latent proteins of the virus up-regulated in EBV+ PTLD_DLBCL the enrichment was notably observed for JAK-STAT, small cell lung and pancreatic-related, chemokine, cytokine-cytokine receptor, Toll like receptor, and apoptosis signaling pathways (Supplementary Figure 2E). On the other hand, potential targets of viral miRNAs suppressed in EBV+ BL were enriched, most remarkably, in cytokine and chemokine-related, JAK-STAT and small cell lung cancer signaling pathways (Supplementary Figure 2F). In addition, probable common target genes of EBV that were suppressed in EBV+ BL and induced in EBV+ PTLD were enriched in chemokine and cytokine, apoptosis and cancer related (small cell lung cancer, pancreatic and prostatic cancers) pathways (Supplementary Figure 2G). No pathway enrichment was found for EBV target genes down-regulated in EBV+ PTLD_DLBCL and up-regulated in EBV+ BL.

Together, these data suggest that EBV modulation of the transcriptional profile of BL and PTLD acts through the differential regulation of relevant cellular programs that are consistent with the status of the host immune system in which lymphomagenesis occurs.

### PTLD_DLBCLs are slightly different based on the EBV latency program

In this study we aimed to investigate the main differences between EBV-associated lymphomas characterized by different expression of EBV-encoded molecules (i.e., diverse latency patterns). To this aim, we used EBV+ BL as prototypic example of latency type I and EBV+ PTLD_DLBCL as example of latency types II/III. As differences emerged between the two diseases apparently related to the differential expression of miRNAs, EBNAs and LMP-1 when PTLD_DLBCL were considered as unique group (the majority of cases displaying a latency type III pattern), we intended to verify the real homogeneity of this tumor.

When studied by unsupervised analysis, all EBV+ PTLD_DLBCLs were mixed up, irrespectively of the latency type (Supplementary Figure 3A). Conversely, when a supervised approach was adopted, we identified 974 probes (equivalent to 623 genes) differentially expressed in EBV+ PTLD_DLBCL latency type II vs. EBV+ PTLD_DLBCL latency type III (Supplementary Data Sheet 13; Supplementary Figure 3B). Based on that, we specifically studied by GSEA the possible contribution of EBV-encoded molecules. As expected, in line with previous results, the two groups showed differences only related to EBNA-2 and EBNA-3s expression; particularly, genes up-regulated upon EBNA-2/EBNA-3s induction were up-regulated in latency type III PTLD_DLBCLs (Supplementary Figure 4). By contrast, no significant differences emerged when EBNA-1, LMP-1, and miRNA related signatures were tested (Supplementary Figures 4, 5).

Finally, in order to challenge the robustness of our results on EBV+ BL (that rely on the comparison with EBV+ PTLD_DLBCL as a unique group) we tested how homogeneous is EBV+ PTLD_DLBCL in comparison to EBV+ BL regarding the EBV signatures. Indeed, by hierarchical clustering we observed that all EBV+ PTLD_DLBCLs, except one case, irrespective of the latency type clustered together in one branch, while EBV+ BL clustered in the other branch, with only one exception (Supplementary Figure 6A). Consistently, when only EBV-driven genes differentially expressed between EBV+ BL and EBV+ PTLD_DLBCL latency type III were considered (Supplementary Data Sheet 14), EBV+ PTLD_DLBCL latency type II clearly clustered within EBV+ PTLD_DLBCLs, being well distinct from EBV+ BL (Supplementary Figure 6B).

Together, these data indicate that, despite some differences mainly related to EBNAs, EBV+ PTLD_DLBCL, independently from latency type, represents a relatively homogeneous group of tumors that can be reliably used as a model against EBV+ BL.

## Discussion

Numerous studies suggest how, by means of its limited but well-evolved “arsenals,” EBV might disrupt and/or recruit physiologic mechanisms of the cell and affect different aspects of cell's life, which might result to its contribution to different malignancies (Thorley-Lawson, 2001; Dirmeier et al., 2003; Altmann and Hammerschmidt, 2005; Barth et al., 2008; Allday, 2009; Babu et al., 2011; Onnis et al., 2012). These arsenals include a set of 9 latent proteins, two non-encoding RNA molecules (EBERs), and a set of miRNAs, belonging to two families called BART and BHRF. However, in different conditions, the virus expresses different combinations of these molecules, which are defined as latency types (De Falco et al., 2009; Camargo et al., 2011; Grinde, 2013; Grywalska et al., 2013).

Latency type I is primarily observed in BL, where only one latent protein (EBNA-1), EBERs and BART microRNAs are expressed (Bellan et al., 2005; Piccaluga et al., 2011; Qiu et al., 2011). On the other hand, the most extreme expression pattern, called latency type III, which includes the expression of all latent proteins, EBERs and, both BART and BHRF miRNAs, is observed in lymphoblastoid cell lines, and a fraction of PTLDs in immunosuppressed individuals (Xia et al., 2008; Morscio et al., 2013a,b). The other type of EBV latency, i.e., type II, which is characterized by the expression of most of EBV latent proteins and BART miRNAs, plus a faint expression of BHRF miRNAs. This latency type is found in a fraction of PTLDs, Hodgkin's Disease Nasopharyngeal Carcinoma and Gastric Carcinoma (De Falco et al., 2009; Qiu et al., 2011).

Although the role of EBV in BL has been debated at some times, several documents indicate the possibility of such a role. The most striking *in vitro* evidence of such role is based on natural selection: despite the possibility of the harboring cells to lose EBV by chance, infected tumor cells sustain it, indicating their dependency upon the virus (Vereide and Sugden, 2009). The model proposed by Niller and Minarovits was the first to make the clear distinction between transformation being the driving force of LCL-like tumors, while anti-apoptosis being the key mechanism for BL-like tumors (Niller et al., 2003, 2004, 2014). Concordantly, it's been recently revealed that BL cells need to maintain the EBV genome, at least to inhibit apoptosis (Vereide et al., 2014). Furthermore, a recent study that sequenced a limited set of genes in primary BL samples found fewer mutations in EBV+ BLs, when compared with EBV- BLs, which could reflect how these mutations might compensate for the anti-apoptotic functions of EBV in some cases of EBV-negative sporadic BL. (Giulino-Roth et al., 2012). Of note, a study performed in Uganda uncovered that antibody titers against EBV are higher in children that will develop BL (De-The et al., 1978).

On the other hand, the substantial participation of EBV in EBV+ PTLD is more accepted: immunosuppressed patients with lower cytotoxic T cell response have a higher rate of circulating EBV and restoration of T cell immune response is a major strategy in treating PTLD (Savoldo et al., 2006; Bollard et al., 2012). EBV+ PTLD cell lines are more sensitive to inhibitors of PI3K/mTOR and HSP90 than EBV-negative lymphoma (Alsayed et al., 2008). Furthermore, EBV might interfere with apoptotic pathways: in EBV+ PTLD, the expression level of proapoptotic protein Bim, a critical regulator of lymphocyte survival, is lower when compared to EBV- PTLDs (Ghigna et al., 2013). In addition, the forced loss of EBV in EBV+ PTLD cell lines might result in death, induced by apoptosis (Vereide and Sugden, 2011).

In the recent years, an increasing usage of high throughput technologies like microarray profiling, and more recently next generation sequencing, has been applied to address the role of EBV in different EBV-related malignancies achieved by comparing the same categories of tumors or tumor-driven cell lines harboring or lacking the virus, or confronting different EBV-related tumors at both levels of gene and miRNA profile (Lenze et al., 2011; Piccaluga et al., 2011; Qiu et al., 2011; Yang et al., 2013). However, to our knowledge, no research has ever dealt with a comparison of EBV+ BL in primary tumors, neither at the level of GEP, nor at the level of miRNA profiling.

In this work, we compared the gene and miRNA expression profiles of EBV+ BL and EBV+ PTLD_DLBCL (the most common subtype of PTLD), aiming to reveal their differences/similarities and exploring how the observed similarities/differences could be related to different EBV latency types, which is of type I and types II/III for the former and latter, respectively. We observed that the two entities might be distinguished by a differentiating molecular signature, and, more importantly, we showed that the EBV-encoded latent proteins and miRNAs can affect the molecular signature of the two tumors. Furthermore, we found that the genes affected by viral products in the two tumors might reflect how the virus might use different/similar and sometimes conflicting strategies when the latency type relies mainly on miRNAs (i.e., type I) or genes/proteins (i.e., types II-III).

When we compared the gene expression profiles, unsupervised analysis could roughly distinguish the two entities, although a supervised clustering could clearly make such discrimination. Further, as this is the first report comparing EBV+ BL and EBV+ PTLD_DLBCL, in order to exclude the influence of genes differentially expressed due to the origin of the two tumors (i.e., BL vs. DLBCL) we also performed the same analysis by filtering the results against the molecular signature of BL (5, 22). Indeed, the same pattern of clustering was observed (Figure 2) (Hummel et al., 2006; Piccaluga et al., 2011).

Similar to GEP, miRNA profiling was far more discriminating in supervised rather than the unsupervised analysis. Interestingly, the major part of differentially expressed miRNAs belonged to those encoded by EBV. In addition, we found a lowered expression of BART cluster miRNAs in EBV+ PTLD_DLBCL, while only one BHRF-1-derived miRNA was found to be induced in this tumor. Furthermore, we observed a higher expression of several cellular miRNAs in EBV+ PTLD_DLBCL, among which well-known oncomiRNA mir-155 was notable.

The lower expression of BART miRNAs in EBV+ PTLD in comparison with EBV+ BL in primary tumors has not been reported by now. Although Qiu et al. reported a down-regulation of BART cluster miRNAs in LCL, when compared to Nasopharyngeal Carcinoma and Gastric Carcinoma (which display latency type II), contrary to our results, they identified a matched pattern of BART miRNAs expression in BL biopsies and LCLs (Qiu et al., 2011). It must be noted, though, that LCLs might not be a good representative for EBV+ PTLD_DLBCL tumor samples in every aspect of them. Interestingly, when, by means of GSEA software, we applied our set of genes deregulated by ebv-miR-BART6-3p to EBV+ PTLD_DLBCL of latency type II vs. EBV+ PTLD_DLBCL of latency type III, we found no significant enrichment in any of the two categories, which might either indicate a similar expression level of at least ebv-miR-BART6-3p (or maybe other EBV-encoded miRNAs), or compensation of the related effects by other virus-encoded products, like EBNA-2, or by differences, e.g., genetic/epigenetic events, in the host cells.

It's generally suggested that BHRF miRNAs are expressed mostly in latency type III (Xia et al., 2008; Pratt et al., 2009; Qiu et al., 2011). However, to our knowledge, the only publication that has addressed the matter in EBV+ PTLD detected the presence of only one single BHRF miRNA, i.e., BHRF-1-2 (Nourse et al., 2012). It must be noted, however, that both the current and the abovementioned analyses used FFPE tissues, and this might have contributed to degradation of other BHRF miRNAs, although this needs to be further verified by miRNA profiling of paired fresh and FFPE samples.

Furthermore, we found that in the absence of cellular miRNAs, viral miRNAs could distinguish the two malignancies in a supervised HCA, which could demonstrate a specific viral miRNA signature for different EBV-related tumors/latency types, as suggested by others (Cai et al., 2006; Pratt et al., 2009; Qiu et al., 2011).

The higher expression of cellular miRNAs in EBV+ PTLD_DLBCL could be explained in two ways, first, it's well accepted that EBV latent proteins might manipulate the expression of cellular miRNAs, as shown in the case of induction of mir-155 by the virus and its lack of expression in primary BL cases (Kluiver et al., 2006; Gatto et al., 2008; Linnstaedt et al., 2010). Second, it's been reported that BART miRNAs might target DICER, a central component of miRNA processing, which probably is a strategy used by the virus to counteract the cell's defense mechanism achieved by anti-viral miRNAs (Iizasa et al., 2010; Skalsky et al., 2012).

It's been known that latent proteins of EBV are able disrupt the cellular gene expression profile, which could be achieved by direct binding to the cell's DNA, as in the case of EBNA-1, or by interaction with different cellular proteins, which seems to be a common phenomenon seen in all EBV latent proteins (Maier et al., 2006; Dresang et al., 2009; White et al., 2010). For example, LMP-1, a homolog of CD40 molecule, is able to activate important signaling pathways related to growth and survival, like JAK-STAT and NF-kB, and thus is considered an oncogene (Chen et al., 2001; Chung et al., 2013); EBNA-1 and EBNA-3C, and the LMP-1 are able to induce genomic instability in different manners (Gruhne et al., 2009); and EBNA-2 interacts with Nur77 to protect against Nur77-mediated apoptosis (Lee et al., 2004).

By means of GSEA, we were able to show how the expression of the genes deregulated by different EBV latent proteins including EBNA-2, EBNA-3s and LMP-1 could be affected in EBV+ PTLD_DLBCL. This is of major interest, since it successfully translates the obtained results of *in vitro* studies in an *in vivo* context, and, more importantly, confirms the manipulation of the molecular state of the tumor cells by EBV. Furthermore, lack of this significant enrichment of EBNA-1 target genes in the two groups might reflect how it might contribute equally to the molecular profile of these tumors. It must be mentioned that we excluded this type of analysis for LMP-2A and -2B, since it has been suggested that LMP-2B regulates the activity of LMP-2A (Rovedo and Longnecker, 2007), but no study so far has addressed the effect of their concomitant expression on the cell's gene expression profile.

Recent publications point at some important roles of BART miRNAs in the pathologic effects exerted by EBV which mostly relate to their anti-apoptotic role in EBV-related tumors (Choy et al., 2008; Dolken et al., 2010; Choi et al., 2013). Particularly, Vereide et al. suggested that BL cell lines depend on BART miRNAs to sustain their malignant characteristics (Vereide et al., 2014). As being concerned with the role of EBV-encoded products, we showed how an EBV-encoded miRNA, i.e., ebv-miR-BART6-3p, could affect the global gene expression signature of EBV+ BL. Aiming this as a proof of principle, it could be deduced that other viral miRNAs might have a similar impact (Ambrosio et al., 2014). It must be noted, however, that the final outcome might not necessarily be simply a sum of the effect of every single miRNA, since it has been proposed that the diseases affected by miRNAs might be a subject of miRNA-miRNA synergism (Riley et al., 2012).

So far, our results indicated that the EBV can change the molecular profile of the tumors, primarily by means of its miRNAs and latent proteins in EBV+ BL and EBV+ PTLD_DLBCL, respectively. We asked, however, for what reason the virus suppresses its BART miRNAs and expresses BHRF cluster in the latter, where all the latent proteins are expressed. We deduced that it might be explained in two ways: first, by the differences in the strategies that the virus uses when it alternates between different latency types, as suggested by Vereide et al. (Vereide and Sugden, 2011), and second, by the fact that in some cases there might be a conflict between miRNAs and latent proteins, or between their physiological effects. For example, the expression of viral latent proteins LMP-1, LMP-2A, and EBNA-2 might be regulated by BART miRNAs, which seems to be a strategy of the virus for controlling its latency programs (Lung et al., 2009; Iizasa et al., 2010, 2012; Riley et al., 2012). In order to test this hypothesis, we tried to determine which genes were specifically deregulated by EBV-encoded products, and what the role of such deregulated genes could be.

When we analyzed the genes potentially regulated by EBV in EBV+ BL or EBV+ PTLD, by either BART miRNAs or latent proteins, they were enriched for different biological processes, which included regulation of apoptosis, gene expression, immune system, and other processes. This finding supported our first hypothesis regarding differences in the mechanisms used by EBV in different latency types. Of note, however, KEGG pathway analysis resulted in enrichment for both different (like Toll Like Receptor) and common (like JAK-STAT) cancer-related pathways.

Of note, the enrichment for cancer-related processes and pathways was higher in genes affected in EBV+ PTLD_DLBCL rather than EBV+ BL. This confirms the previous findings of Vereide et al. which discovered higher dependency for EBV in EBV+ PTLD cells, when compared to BL cells (Vereide and Sugden, 2011).

Inspired by the latter results, we asked if there were any common genes between those potentially suppressed in EBV+ BL and those likely induced in EBV+ PTLD_DLBCL that would support our second hypothesis. Interestingly, we found 38 genes with such characteristics that were enriched for cancer-related processes and pathways.

This finding, as we suggest, gives another explanation for which EBV turns down its BART miRNAs in EBV+ PTLD_DLBCL, where the entire set of latent proteins is expressed: there might be a conflict between some BART miRNAs and latent proteins in the modes that they influence the cellular processes, i.e., some of the target genes that are suppressed by viral miRNAs might be induced by viral latent proteins. Furthermore, as mentioned above, there might be a similar contradiction in the way the virus might affect cellular miRNAs. Thus, simultaneous expression of these two different categories of products might cost the virus loss of control on its latency state, or in extreme conditions, its ability to successfully infect the cells and produce virions.

Collectively, our results suggest that in different malignancies harboring different latency types of EBV, this virus might exert a role by means of different and maybe conflicting mechanisms, which could explain differential expression of its latent proteins and miRNAs. However, these results need to be further confirmed by functional analysis experiments. Furthermore, our current understanding of the role of EBV in human tumors is mostly based on *in vitro* experiments. In this regard, analysis of EBV-related primary tumors by means of massive parallel sequencing methods might help us to improve our knowledge of the manifestation of the virus *in vivo* and probably might result in specific EBV-targeting therapies.

## Financial support

This work was supported by the Centro Interdipartimentale per la Ricerca sul Cancro “G. Prodi,” BolognAIL, AIRC 10007 5xMille—Prof. Pileri, AIRC IG 2013 N.14355—Prof. Piccaluga, RFO (Prof. Pileri and Prof. Piccaluga), Progetto Strategico di Ateneo 2006 (Prof. Piccaluga), and FIRB Futura 2011 RBFR12D1CB (Prof. Piccaluga).

## Conflict of interest statement

The authors declare that the research was conducted in the absence of any commercial or financial relationships that could be construed as a potential conflict of interest.
