# Supplementary material for: Molecular signature of Epstein Barr virus-positive Burkitt lymphoma and post-transplant lymphoproliferative disorder suggest different roles for Epstein Barr virus
Source: Front Microbiol. 2014 Dec 23;5:728. doi: 10.3389/fmicb.2014.00728 (PMC4274971; doi:10.3389/fmicb.2014.00728)
Supplement: Supplementary file 15 [file Presentation1.PDF]

Supplementary Material

Molecular signature of Epstein Barr virus-positive Burkitt lymphoma and post transplant lymphoproliferative disorder suggest different roles for Epstein Barr virus

Running Title: EBV-encoded miRNA and genes in NHL

Mohsen Navari[1-3], Fabio Fuligni[1], Maria Antonella Laginestra[1], Maryam Etebari[1], Maria Raffaella Ambrosio[2], Maria Rosaria Sapienza[1], Maura Rossi[1], Giulia De Falco[2&4], Davide Gibellini[5], Claudio Tripodo[6], Stefano A. Pileri[1], Lorenzo Leoncini[2], Pier Paolo Piccaluga[1]*.

[1]Hematopathology Section, Department of Experimental, Diagnostic, and Experimental Medicine, S. Orsola-Malpighi Hospital, Bologna University School of Medicine;
[2]Department of Medical Biotechnology, University of Siena, Italy;
[3]Department of Basic sciences, Torbat Heydariyeh University of Medical Sciences, Torbat Heydariyeh, Iran.
[4]Nanchang Joint Programme in Biomedical Sciences, School of Biological and Chemical Sciences, Queen Mary University of London, UK
[5]Microbiology Section, Department of Experimental, Diagnostic, and Experimental Medicine, S. Orsola-Malpighi Hospital, Bologna University School of Medicine;
[6]Tumour Immunology Unit, Department of Health Science, Human Pathology Section, Palermo University School of Medicine, Italy.

***Correspondence:**
Pier Paolo Piccaluga, MD, PhD
Hematopathology Section, Department of Experimental, Diagnostic, and Experimental Medicine, S. Orsola-Malpighi Hospital, Bologna University School of Medicine;
Via Massarenti, 9 - 40138 Bologna, Italy.
Phone: 0039-051-6364043; Fax: 0039-051-6364037;
e-mail: pierpaolo.piccaluga@unibo.it

1. Supplementary Figures and Tables.

1.1. Supplementary tables

Supplementary Data Sheet 1: sample description of PTLD cases used in Gene Expression Profiling Analysis. The data were kindly provided by Prof. Thomas Tousseyn and R. Julie Morscio (University of Leuven). Abbreviations: **M**, Male; **F**, Female; **GALT**, Gut-associated Lymphoid Tissue; **Waldeyer**, Waldeyer's tonsillar ring; **LN**, Lymph Node; **BALT**, Bronchus-associated lymphoid tissue; **DLBCL**, Diffuse Large B Cell Lymphoma; **PTLD**, Post Transplant Lymphoproliferative Disorder; **M-B-PTLD,** Monomorphic B-cell Post Transplant Lymphoproliferative Disorder.

Supplementary Data Sheet 2: sample description of BL and PTLD cases used in miRNA Expression Profiling Analysis.

Supplementary Data Sheet 3: Genes differentially expressed between EBV+ BL and EBV+ PTLD_DLBCL.

Supplementary Data Sheet 4: Predicted targets for viral miRNAs.

Supplementary Data Sheet 5: List of deregulated genes upon transfection of kata 2A8 cell line with ebv-miR-BART6-3p mimic.

Supplementary Data Sheet 6: List of deregulated genes upon transfection of Akata cell line by ebv-miR-BART6-3p inhibitor.

Supplementary Data Sheet 7: List of genes regulated by ebv-miR-BART6-3p.

Supplementary Data Sheet 8: Candidate viral latent protein targets up-regulated in EBV+ PTLD_DLBCL.

Supplementary Data Sheet 9: Candidate viral latent protein targets down-regulated in EBV+ PTLD_DLBCL.

Supplementary Data Sheet 10: Candidate viral miRNA targets up-regulated in EBV+ BL.

Supplementary Data Sheet 11: Candidate viral miRNA targets down-regulated in EBV+ BL.

Supplementary Data Sheet 12: List of genes potentially up-regulated in EBV+ PTLD_DLBCL by EBV-encoded latent proteins AND potentially down-regulated in EBV+ BL by EBV-encoded miRNAs.

Supplementary Data Sheet 13. Genes differentially expressed between EBV+ PTLD_DLBCL Latency type III and EBV+ PTLD_DLBCL Latency type II.

Supplementary Data Sheet 14. EBV signature-related genes genes differentially expressed between EBV+ PTLD_DLBCL Latency type III and EBV+ BL

1.2. Supplementary Figures

Supplementary Figure 1. Genes regulated by viral latent proteins and miRNAs are significantly over-represented in EBV+ BL and EBV+ PTLD_DLBCL. Genes up-regulated by viral latent oncoproteins (EBNA-2, EBNA-3s, and LMP-1) are over-represented among genes over-expressed in EBV+ PTLD_DLBCL vs. EBV+ BL (A). Similarly, genes down-regulated by viral latent oncoproteins (EBNA-2, EBNA-3s, and LMP-1) are over-represented among genes down-regulated in EBV+ PTLD_DLBCL vs. EBV+ BL (B). BART family miRNAs "targets" (including both genes down-regulated upon ebv-miR-BART6-3p transfection or up-regulated upon ebv-miR-BART6-3p inhibition in this study as well as all target genes predicted by bioinformatics) were over-represented among genes down-regulated in EBV+ BL vs. EBV+ PTLD_DLBCL (C). Consistently, genes up-regulated upon ebv-miR-BART6-3p transfection and down-modulated upon its inhibition were over-represented among genes up-regulated in EBV+ BL vs. EBV+ PTLD_DLBCL (D). Interestingly, genes down-regulated by ebv-BART6-3p in EBV+ BL and those up-regulated by viral latent oncoproteins in EBV+ PTLD_DLBCL were overlapping (E).

Supplementary Figure 2. EBV-deregulated genes in different EBV latency types might interfere with relevant biological processes. MSigDB tool was used to identify biological processes, as defined by GeneOntology, significantly over-represented among genes whose expression was affected by EBV molecules. A: genes up-regulated by EBV latent proteins; B: genes down-regulated by EBV latent proteins; C: genes suppressed by EBV-miRNA; D: genes xuppressed by EBV-miRNA and Induced by EBV latent proteins. MSigDB tool was then used to identify pathways, as defined by KEGGpath, significantly over-represented among genes whose expression was affected by EBV molecules: E: genes up-regulated by EBV latent proteins; F: genes suppressed by EBV-miRNAs; G: genes suppressed by EBV-miRNA and induced by EBV latent proteins.

Supplementary Figure 3. Two subtypes of EBV+ PTLD_DLBCL are slightly different based on the EBV latency program. A) Unsupervised analyses based on gene expression failed to distinguish the two PTLD_DLBCL subtypes (defined according to the EBV latency types: II Vs. III). B) Supervised hierarchical clustering based on the differentially expressed genes is shown. In the heat-map each row represents a gene (A) and each column represents a sample.

The color scale illustrates the relative expression level of a gene across all samples: red represents an expression level above the mean, green represents expression lower than the mean.

Supplementary Figure 4. Gene Set Enrichment Analysis (GSEA) of EBV latent proteins-dependent signatures in EBV+ PTLD_DLBCL. The effect of viral latent proteins expressed across the different latency programs was investigated in EBV+ PTLD_DLBCL characterized by latency types II or III. GSEA was applied to study the expression of target genes of the latent proteins of EBV in the two tumor subtypes. For each latent protein (EBNA-1, EBNA-2, EBNA-3s, and LMP-1) induced or suppressed targets were analyzed (left and right, respectively). As expected by the latency program, only EBNA-2 and EBNA-3stargets appeared to be significantly enriched in either one group. Particularly, induced target were enriched in latency type III cases.

Supplementary Figure 5. Gene Set Enrichment Analysis (GSEA) of EBV miRNA-dependent molecular signatures in EBV+ PTLD_DLBCL. The effect of viral-encoded miRNA was investigated in EBV+ PTLD_DLBCL characterized by latency types II or III. GSEA was applied to study the expression of ebv-miR-BART6-3p signature in the two tumor subtypes. The genes having a negative correlation with ebv-miR-BART6-3p expression (i.e. the genes induced upon inhibition of the miRNA or suppressed following its expression) or having a positive correlation with ebv-miR-BART6-3p expression (i.e. the genes down-regulated upon inhibition of the miRNA or up-regulated following its expression) were analyzed (left and right, respectively). None of the two groups of the genes were significantly enriched in any of the two tumor entities.

Supplementary Figure 6. Two subtypes of EBV+ PTLD_DLBCL are distinct from EBV+ BL based on EBV-dependent signature independently from the latency pattern. A) Supervised hierarchical clustering based on the entire EBV dependent molecular signature (i.e. the signature composed by all targets of EBNAs, LMPs, and miRNAs). B) Supervised hierarchical clustering based on genes belonging to the EBV dependent molecular signature and differentially expressed between latency type I (i.e. EBV+ BL) and latency type III (i.e. a fraction of EBV+ PTLD_DLBCL). In the heat-map each row represents a gene (A) and each column represents a sample. The color scale illustrates the relative expression level of a gene across all samples: red represents an expression level above the mean, green represents expression lower than the mean.
