# Supplementary figures and images for: Molecular signature of Epstein Barr virus-positive Burkitt lymphoma and post-transplant lymphoproliferative disorder suggest different roles for Epstein Barr virus
Source: Front Microbiol. 2014 Dec 23;5:728. doi: 10.3389/fmicb.2014.00728 (PMC4274971; doi:10.3389/fmicb.2014.00728)

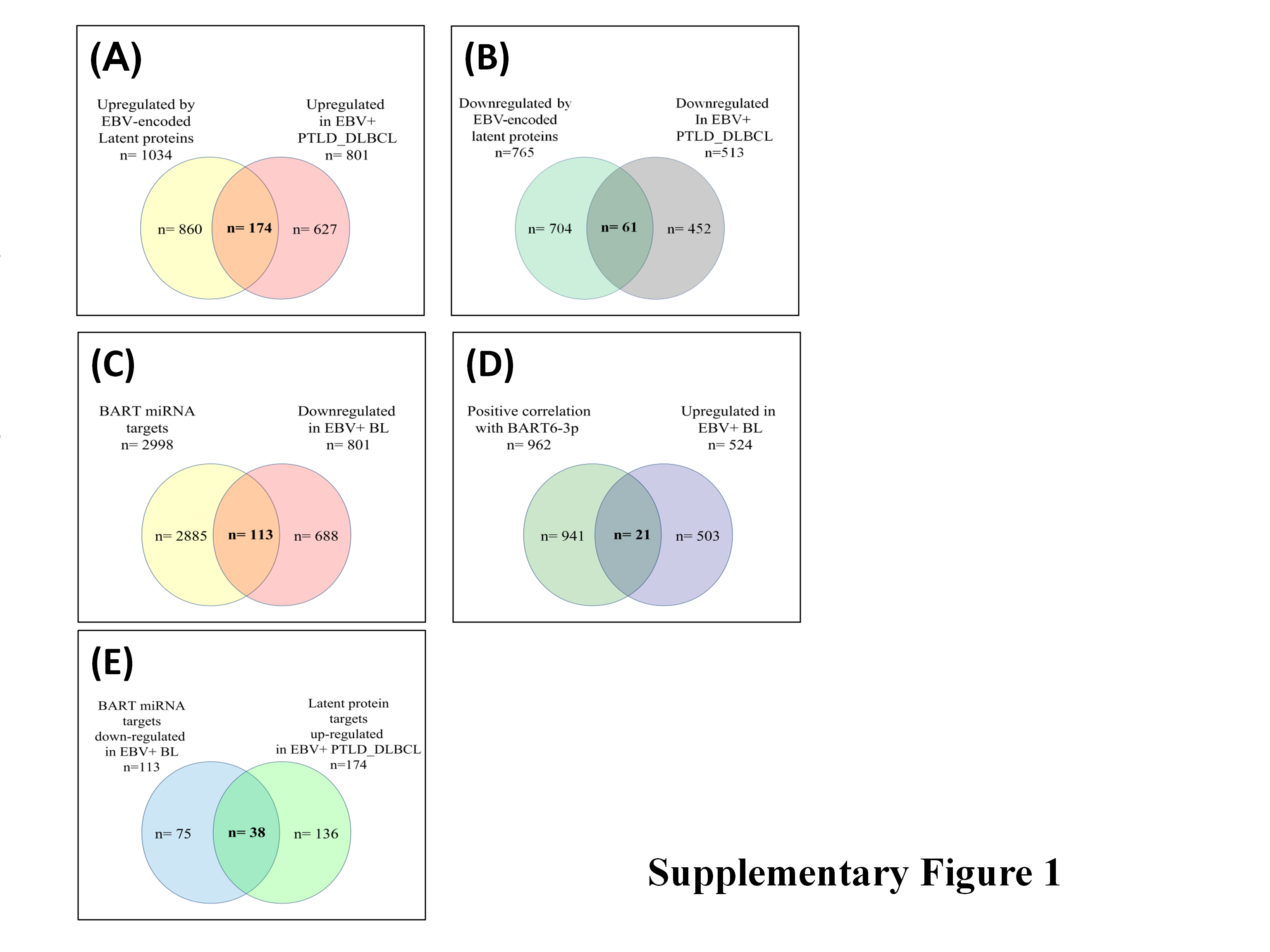

Supplement: Supplementary file 16 [file Image1.TIF]

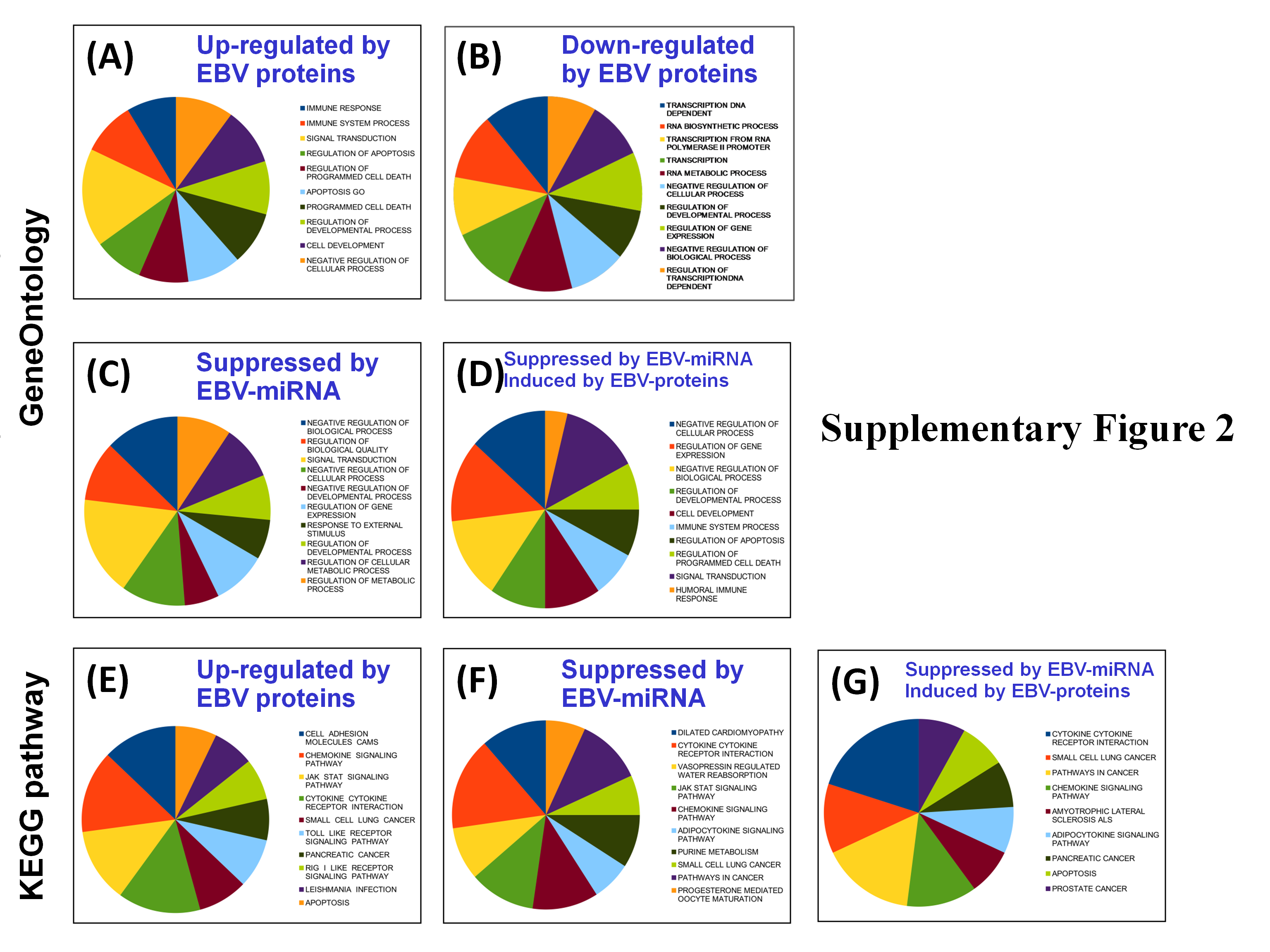

Supplement: Supplementary file 17 [file Image2.TIF]

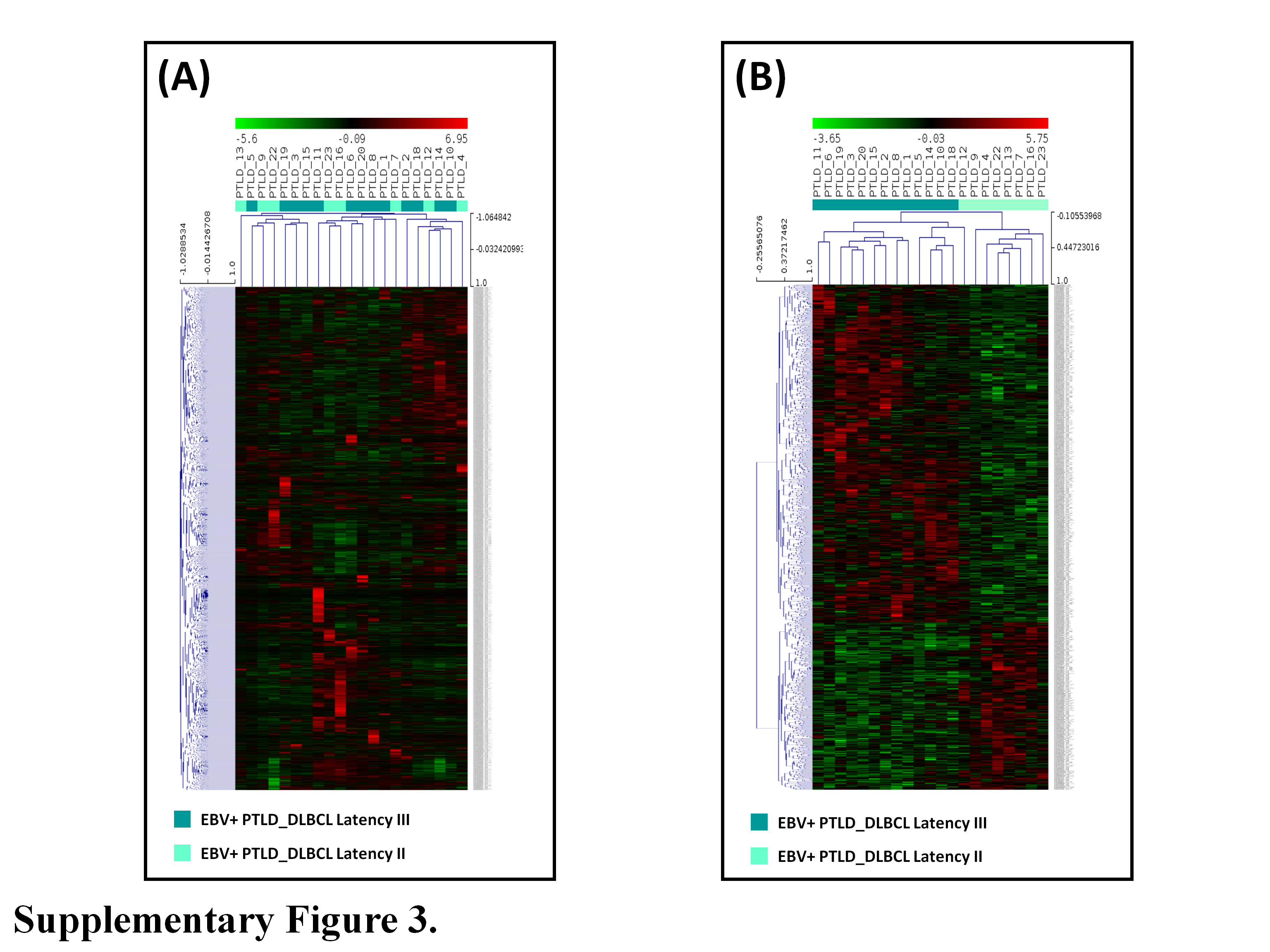

Supplement: Supplementary file 18 [file Image3.TIFF]

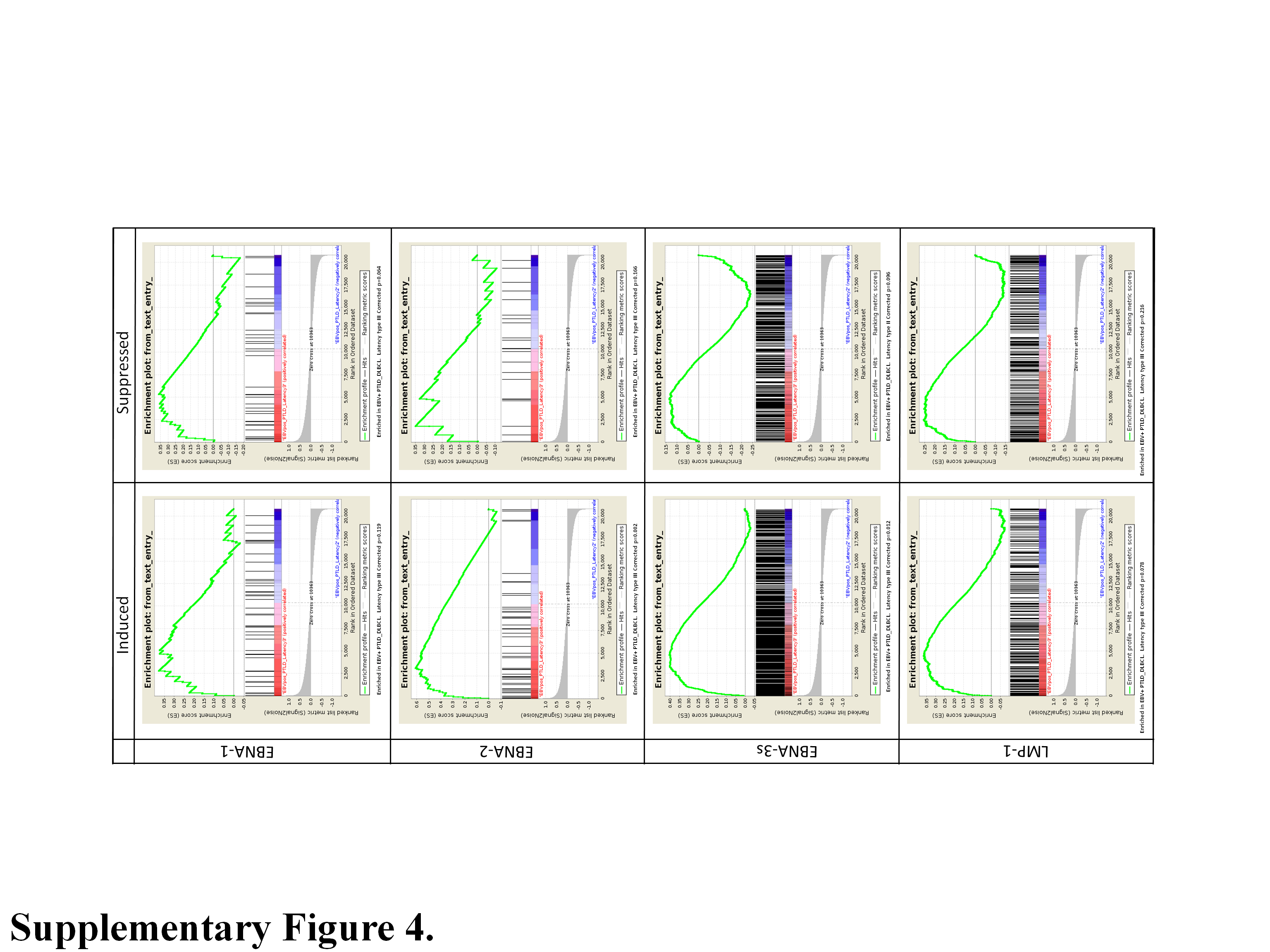

Supplement: Supplementary file 19 [file Image4.TIFF]

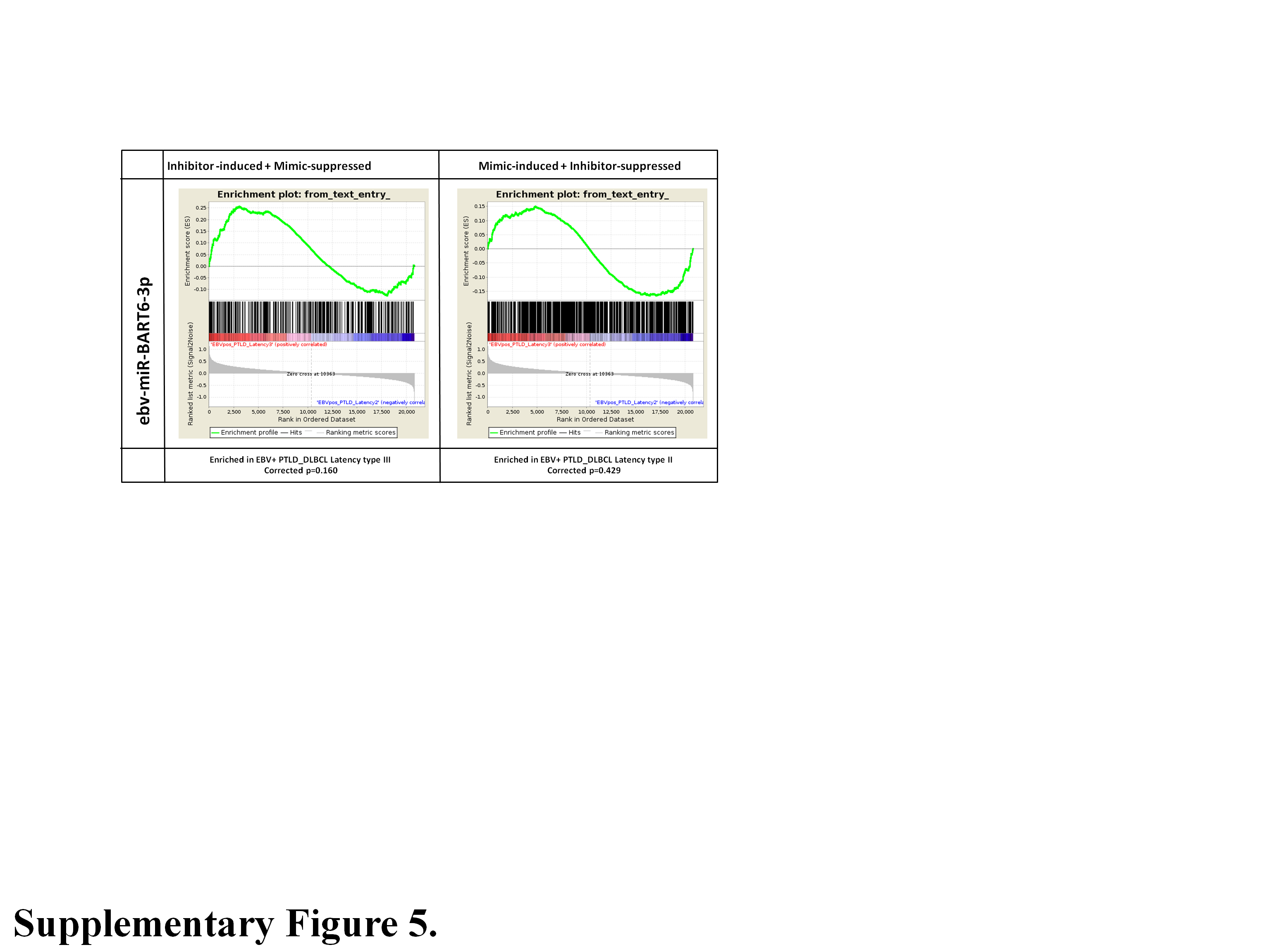

Supplement: Supplementary file 20 [file Image5.TIFF]

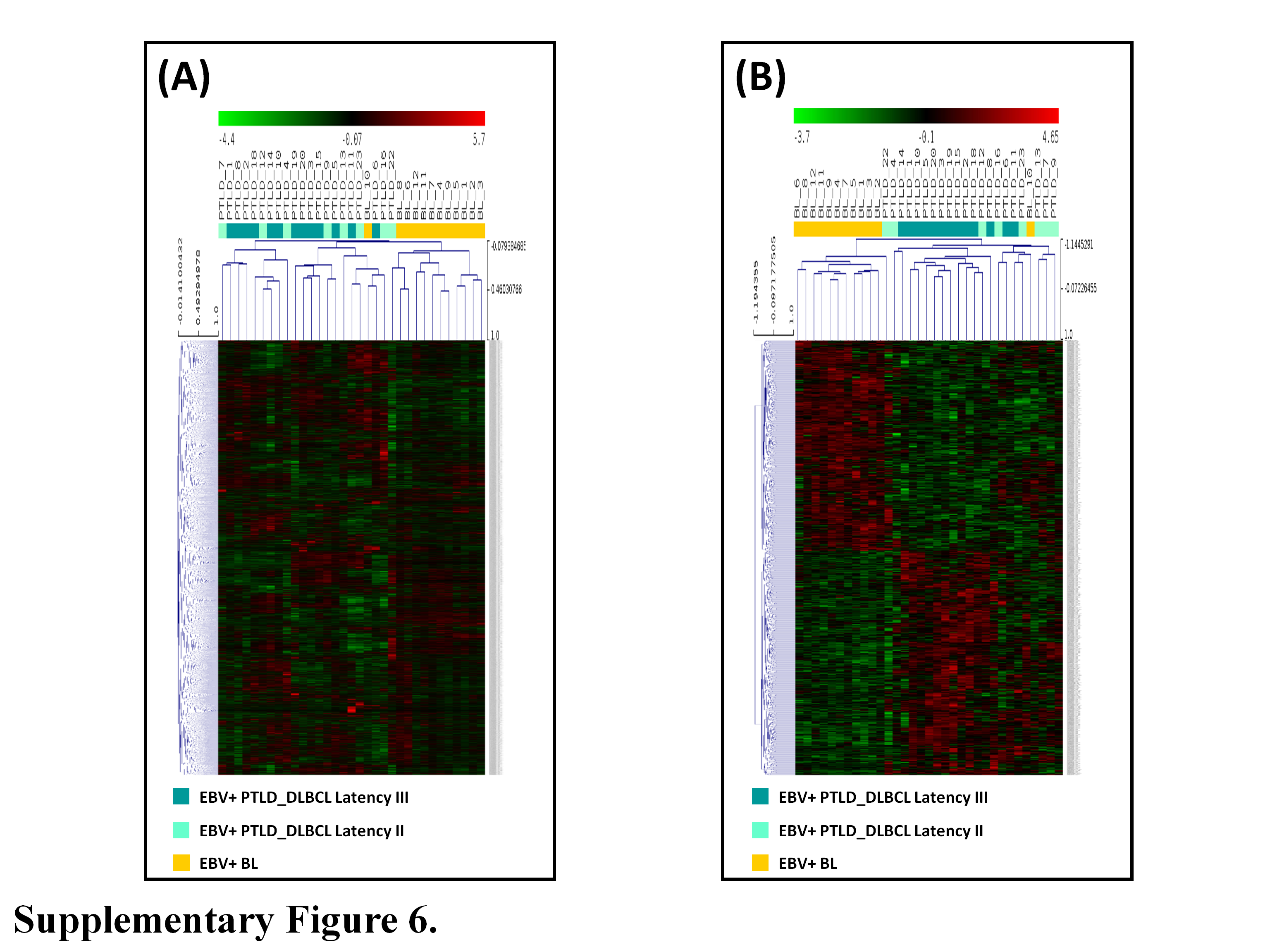

Supplement: Supplementary file 21 [file Image6.TIFF]
